# Insertion Process of Ceramic Nanoporous Microneedles by Means of a Novel Mechanical Applicator Design

**DOI:** 10.3390/pharmaceutics7040503

**Published:** 2015-11-18

**Authors:** Xavier H. M. Hartmann, Peter van der Linde, Erik F. G. A. Homburg, Lambert C. A. van Breemen, Arthur M. de Jong, Regina Luttge

**Affiliations:** 1 Department of Mechanical Engineering, Microsystems Group, Materials Technology Institute (MaTe) and ICMS Institute for Complex Molecular Systems, Eindhoven University of Technology, Den Dolech 2, 5612 AZ Eindhoven, The Netherlands; E-Mails: x.h.m.hartmann@student.tue.nl (X.H.M.H.); f.g.a.homburg@tue.nl (E.F.G.A.H.); 2 MESA+ Institute for Nanotechnology, University of Twente, 7500 AE Enschede, The Netherlands; E-Mail: p.vanderlinde@utwente.nl; 3 MyLife Technologies B.V., 7522 NH Enschede, The Netherlands; 4 Department of Mechanical Engineering, Polymer Technology Group and Materials Technology Institute (MaTe), Eindhoven University of Technology, P.O. Box 513, 5600 MB Eindhoven, The Netherlands; E-Mail: l.c.a.v.breemen@tue.nl; 5 Department of Applied Physics, Molecular Biosensing for Medical Diagnostics and ICMS Institute for Complex Molecular Systems, Eindhoven University of Technology, P.O. Box 513, 5600 MB Eindhoven, The Netherlands; E-Mail: a.m.de.jong@tue.nl

**Keywords:** ceramic nanoporous microneedles, soft lithography, micromolding, (trans)dermal drug delivery, microneedle patch, thumb pressure, applicator, skin insertion, fracture force

## Abstract

Arrays of microneedles (MNAs) are integrated in an out-of-plane fashion with a base plate and can serve as patches for the release of drugs and vaccines. We used soft-lithography and micromolding to manufacture ceramic nanoporous (np)MNAs. Failure modes of ceramic npMNAs are as yet poorly understood and the question remained: is our npMNA platform technology ready for microneedle (MN) assembly into patches? We investigated npMNAs by microindentation, yielding average crack fracture forces above the required insertion force for a single MN to penetrate human skin. We further developed a thumb pressure-actuated applicator-assisted npMNA insertion method, which enables anchoring of MNs in the skin by an adhesive in one handling step. Using a set of simple artificial skin models, we found a puncture efficiency of this insertion method a factor three times higher than by applying thumb pressure on the npMNA base plate directly. In addition, this new method facilitated zero MN-breakage due to a well-defined force distribution exerted onto the MNs and the closely surrounding area prior to bringing the adhesive into contact with the skin. Owing to the fact that such parameter space exists, we can conclude that npMNAs by soft lithography are a platform technology for MN assembly into a patch.

## 1. Introduction

Adopting miniaturization technology has been key to successfully applying novel devices in the life-sciences industry. Such miniaturized devices are hence also explored for the manipulation of tiny amounts of fluids, consequently coined microfluidics. This fairly new research discipline now presents us with the possibility of fabricating microneedle arrays for use in drug delivery *via* the skin. In due course of the last decade, an assortment of microneedle designs that vary tip shape, length, diameter, as well as density of the needles within the array and its material were demonstrated and several commercial activities were already established [1,2,3,4]. Significant pre-clinical progress has been made to evaluate these different microneedle platform technologies for their function, including (a) efficiency of skin breaching, (b) understanding cargo release mechanisms, and (c) control of the cargo release profiles from the device into the skin. However, for many design varieties of microneedles presented in the scientific literature so far, clinical evaluation for transdermal drug delivery is on-going. To bring any of these minimally invasive devices to market-entry, one has to develop suitable models that allow clinical researchers to benchmark these emerging microneedle techniques not only against the state of the art, *i.e.*, the hypodermic needle, but also amongst each other to define the best possible method of drug delivery for a specific medical need.

It is the objective of this work to assess whether ceramic nanoporous microneedle arrays (npMNAs) could be assembled into a patch for further usage in drug administration. We have already established that ceramic npMNAs can serve as such a new platform technology for (trans)dermal delivery of drugs and vaccines [5,6]. Therefore, the focus of this study is on the insertion process by means of a novel mechanical applicator design.

It is the general understanding that the many developed manufacturing processes for microneedles (MNs) result in distinct device properties to be utilized in patch technology for drug delivery via the skin [7,8,9,10,11]. The fabrication process of our ceramic npMNAs has been previously described by us in detail [12].

In this paper, we explain the engineering design process of the novel mechanical applicator. It is capable of inserting and anchoring individual MN tips of an npMNA in the skin in conjunction with an adhesive patch, which is a preferred embodiment of such a delivery system. The mechanical boundary conditions of the insertion process will influence drug delivery performance, specifically when diffusion requiring prolonged delivery times (minutes to hours) is the main mechanism of delivery, as in our case [6]. Consequently, the boundary conditions must be controlled during insertion of the microneedles.

Previously, ceramic npMNAs, which were applied with thumb-pressure only, yielded variable release efficiencies of a few percent in human skin [5], we hypothesized that microneedle insertion efficiency may be low, too, or at least highly variable due to insufficient force applied. Impact insertion of the same type of npMNAs instead showed an insertion efficiency of greater than 80%. Therefore, also for the evaluation of our novel engineered design of a thumb-pressure actuated mechanical applicator, we apply ceramic npMNAs; hence, this paper focuses on the performance study of this novel insertion process by means of three simplified and cost-efficient artificial models of the skin.

Several excellent review papers published elsewhere [13,14,15,16,17] on the transdermal delivery of drugs with MNs exist, including the one by Ita recently presented in this issue [13]. Aside from a more general overview of different types of MNs and their prospective utility in pharmaceutics, Ita provides us with an insight into the recent trend in MNs to capitalize on advanced functional materials, but not without a critical note on the challenges that remain in bringing these devices into the clinic.

Overall, MNA technology lacks a thorough analysis of their assembly into a wearable patch being self-administered by the user. Addressing these technical issues scientifically will provide further evidence of their applicability and motivate the introduction of such technologies to the market.

From the literature so far, we conclude that the three main issues related to MNA insertion methods are: (1) Lack of characterization of the exact MN geometry (tip radius, tip shape, including tip length, and resulting total length in the case of MN tips extended by a shaft) and its interplay with the skin upon an applied load; (2) Lack of characterization of other needle array parameters such as array density, influence of base plate diameter *versus* the effective needle array area and the resulting force distribution in the interplay of the device with a soft and complex material such as skin; (3) Lack of knowledge of material properties, either of the skin or of the device itself. These three issues hamper the further development of a benchmark strategy for the great many different MNA technologies, even if the same type of vaccine, drug, or model marker (*e.g.*, nanoparticles or fluorescent dyes) is applied by the different MNAs in a delivery performance test. Hence, most researchers, including us, simply compared their own novel MNA technology to the gold standard in drug delivery technology; that is, the hypodermic needle either by intradermal, subcutaneous, or intramuscular injection [5,18,19,20,21].

To make a step forward in technically assessing MNA performance for their specific delivery purpose as a self-administered patch, we fist carried out desk research by accessing commonly available databases (*Scopus)* based on the combination of two key-words in the search: (1) microneedles, and (2) self-administration [22]. We only found 19 papers, of which just four papers actually addressed these two key-words in the article abstract within the context of a method of MNA insertion [23,24,25,26]. We took the latter as a hint in assuming that the authors of these four articles also explained their method of insertion carefully. Based on this straightforward assumption, we studied their papers with a focus on the effectiveness of MNA insertion, which is required for self-administered patches.

Park *et al.*, for example, described biodegradable polymer MNs that encapsulate a drug cargo in the needle matrix, which lowers the fracture force of this type of MNs [23]. The researchers recorded stress-strain curves by applying a commercial force test station. To perform this type of mechanical characterization, they pressed an MNA with 35 MNs against a stainless steel surface. The individual MNs in the study [23] were described with a base radius of 100 µm, a total length of 1 mm, and a tip radius of 12 µm, which Park *et al.* considered to be relatively blunt. They selected a displacement velocity of 1.1 mm/s and recorded the data until a maximum load of 19.6 N was reached. However, an abrupt fall in the applied force values indicated failure of the MNs. They visually inspected the MNs and found that all MNs deformed and failed uniformly. On the other hand, they also found that similar MNs can be actually inserted into human skin with a force as low as 45 mN per needle. Minimal failure force in their technology relates to the amount of drug being encapsulated. In the case of 10% of drug encapsulation failures, forces were as low as 40 ± 2 mN per needle, so these MNs fall short with respect to their own value defined for the insertion force into human skin. The paper by Park *et al.* [23] also discussed that the exact failure force values will highly depend on the needle geometry (length, tip radius, *etc.*). Unfortunately, these details were not further specified in relation to the method of insertion. Here, we can use the insertion force value as a guideline in our own design process. Without going into all details of their research, we can conclude that the minimally required value of failure force should be given as a multiple of the skin insertion force measured for a specific needle geometry in a defined skin model. A safety factor of 3.6, suggested by Park *et al.*, will subsequently lead to a minimally required fracture forces of 162 mN with an estimated insertion force of 45 mN [23].

In the same group, Lee *et al.* put a dissolving MN patch for the transdermal delivery of human growth hormone to the test [24]. These dissolving MNs have another distinguished needle geometry compared to their previous MNA technology and were made by a variant of a soft-lithographic replication process. Lee *et al.* specifically stated the capability of self-administration of this MNA patch by manual insertion. However, the paper gives no specific description of further details of their MN patches in controlling mechanical boundary conditions when applying thumb pressure. Donnelly *et al.* also investigated thumb pressure insertion of a hydrogel-forming MNA patch recruiting 20 healthy volunteers [25]. Also without revealing mechanical details, the authors claimed effective insertion into the skin by self-application without the aid of an applicator device, too. Yet another paper by the Prausnitz group in 2014, by Norman *et al.*, actually described the benefits of using an applicator for MN-patch self-vaccination [26]. Norman *et al.* utilize their patch in combination with a snap-device for self-vaccination against influenza without any specific anchorage of the individual MNs in the skin. However, the authors also conclude that amongst a range of other validation tests, there is a need to improve MN patch administration to reach a reliable insertion of 100% on the first attempt.

Although great progress on the applicability of MNA patches for medical intervention has been achieved lately, we are too limited in our knowledge to link MNA performance with the mechanical boundary conditions during insertion of MNs into the skin. Hence, besides the literature study presented above, we experimentally studied the npMNA insertion process by means of engineering a controlled applicator-skin interface. We also evaluate fracture forces of our ceramic MNs by indentation.

In summary, MNAs are devices that are integrated in an out-of-plane fashion with a base plate. These devices can serve as patches for the release of drugs and vaccines. Previously, our ceramic npMNAs were classified as poke and diffuse approach [6]. Unfortunately, failure modes of ceramic npMNAs are poorly understood and the question remains: is our npMNA a platform technology ready for MN assembly into a patch? To address this question, we performed indentation studies and measured crack fracture forces yielding average values of 1.823 ± 0.455 and 1.341 ± 0.443 N for two respective types of ceramic npMNA. We further developed an applicator-assisted MNA insertion method actuated by thumb pressure, which generated a defined force distribution exerted onto the base plate of the MNA and the closely surrounding area prior to bringing an adhesive layer (containing the npMNA) into contact with the skin. We studied the puncture efficiency in aluminum foil by back-light optical microscopy for different artificial skin models. Our findings confirmed that by using the applicator the puncture efficiency enhanced by a factor of 3 compared to insertion by thumb pressure onto the npMNA base plate directly. In addition, this new insertion method facilitates zero MN-breakage and offers the possibility of anchoring the MNs in the skin by an adhesive patch which is neatly placed onto the skin in one step of handling with the insertion of the MNs. Due to the fact that such parameter space exists, we can conclude that ceramic npMNAs by soft lithography are a platform technology for MN assembly into a patch in conjunction with an appropriate applicator device. In this paper, we discuss the applicator engineering design process and the evaluation of this novel thumb pressure-actuated applicator-assisted MNA insertion method.

## 2. Experimental Section

### 2.1. Materials

**Figure 1:**
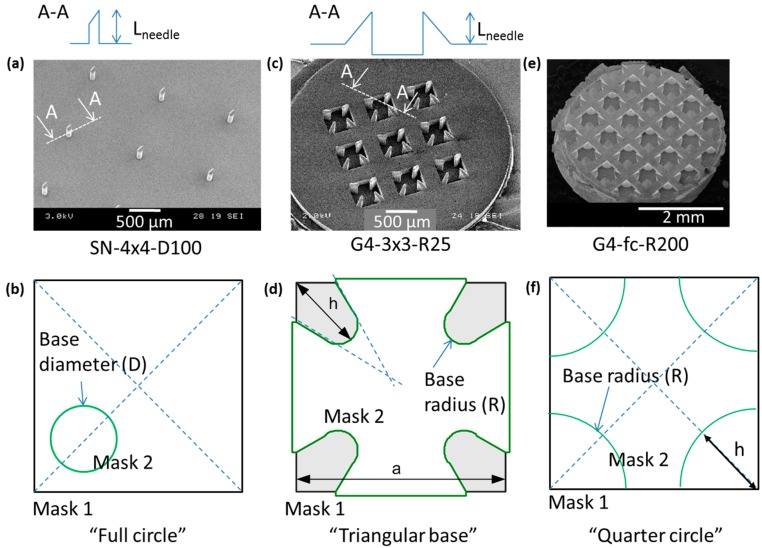
Cross-sectional schematic of the needle shapes, SEM images and mask designs of different nanoporous microneedle arrays (npMNAs) configurations. (**a**,**b**) Single Needle (SN) 4 × 4 npMNA with a base diameter (D) of 100 µm and a needle length (*L*_needle_) of 320 µm; (**c**,**d**) Triangular base groups-of-four (G4) MNs defined in three rows and columns with a microneedle (MN) base radius (*R*) of 25 µm and a needle length (*L*_needle_) of 200 µm; (**e**,**f**) Quarter circle G4 MNs with a MN base radius (*R*) of 200 µm and a needle length (*L*_needle_) of 200 µm fully covering the base plate; the cross-sectional schematic impression would be the same as given in (**c**).

We used ceramic nanoporous microneedle arrays (npMNA) kindly provided by MyLife Technologies BV (Enschede, The Netherlands). All npMNAs were manufactured by a patented fabrication process developed at University of Twente [27], of which the principles of the micro-fabrication technology were reported by us previously [12]. In brief, aluminum oxide nanoparticles were mixed with polymeric binder to obtain a slurry, which were dispensed into a micromachined mold from poly(di)methyl siloxane (PDMS) containing the inverted shape of the microneedles. After drying the green state ceramic part was removed from the mold and disks of a desired diameter were punched out of the green body. The disks act as a base plate which are seamlessly integrated with the microneedles protruding perpendicular from the surface. Subsequently the disks were sintered at 1450 °C, and after polymeric binder decomposition, a strong and nanoporous ceramic npMNA device remains. Final physical dimensions of an npMNA depend on the lithographic process and the shrinkage in the ceramic material during sintering; hence, here we state dimensions by design values only. We used a variety of different npMNA configurations in our experiments (Figure 1a,c,e). One type of design (Figure 1a,b) is a uniformly distributed array of single needles (SN) with a full circle base of a diameter (D) of either 100 or 300 µm and MN length of 320 and 560 µm, respectively. The SNs can be freely arranged in the mold such that they either fully cover (fc) the base plate of the npMNA or with a fixed number of rows and columns (N × N). Another type of design consists of repetitive groups of four (G4) MNs enclosing a cavity (Figure 1c,d,e,f). In this configuration, MN tip direction alternates on a regular grid structure with an inter-cavity spacing of 0.85 mm (Figure 1c,e). The MN tip geometry in this G4-design can be selected either with a triangular base defined by a base radius (*R*) of 25 µm (Figure 1c,d) or with a quarter circle base radius (*R*) of 200 µm (Figure 1e,f). Again, the G4-MNs are either arranged with a dedicated number (3 × 3) of rows and columns (Figure 1c) or fully covering (fc) the base plate (Figure 1e). All the G4-designs used here are designed in the mask with a needle length of 200 µm. Based on this unit cell design the MN density may be defined as 9 MNs/mm^2^, which scales to a few hundred of MNs at full-coverage (fc) for a typical base plate diameter of 9 mm in a patch application. In view of our earlier work on human skin penetration and release studies [5,6], we used this G4-fc-R200 design for the development of a method to measure fracture forces for an individual nanoporous needle in the array. Subsequently, we used a variety of npMNAs with different configurations for the development of our new applicator-assisted insertion method.

### 2.2. Methods

#### 2.2.1. Fracture Force Measurements

**Figure 2:**
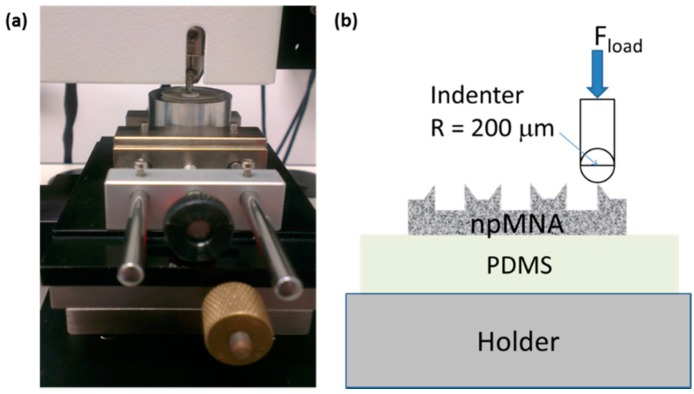
(**a**) CSM microindenter setup showing the sample holder and clamping conditions during indentation; (**b**) Schematic overview of the ceramic npMNA resting on a PDMS rubber pad during application of the load *via* the microindenter.

A microindenter (CSM Instruments) was used for mechanical characterization of MNs. The CSM indenter can exert a normal force on a pre-specified location with a maximal load of 30 N. We used a spherical indenter with a tip radius of 200 μm to apply the load directly onto the tip of a single MN in the array. To avoid unintentional breakage of the base plate of the npMNA due to a slight warp of the base plate or dust particles, and to avoid gliding of the npMNA on the sample holder, we placed a polydimethylsiloxane (PDMS) rubber pad of 10 × 10 mm square and 0.9 mm thickness between the npMNA and the sample holder of the tester. PDMS was mixed in a 10:1 ratio such as it is commonly applied for the fabrication of microfluidic devices [28]. This type of PDMS has a tunable Young’s modulus with a typical value of ~750 kPa. Figure 2 depicts the measurement set-up.

#### 2.2.2. Applicator Fabrication and Design

**Figure 3:**
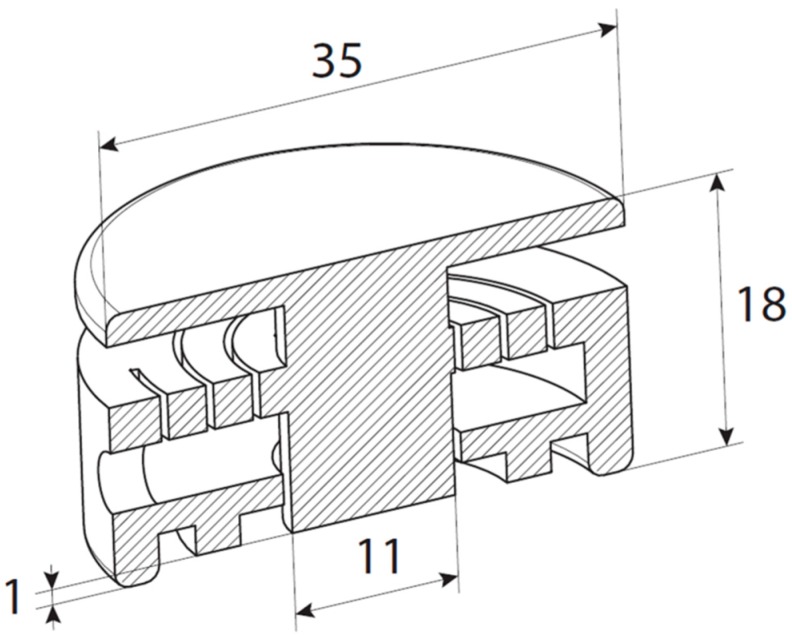
Technical drawing of the mechanical applicator (all dimensions at scale in mm).

For an initial design study of the applicator-assisted insertion method, we used in-house CO_2_ laser cutting for the fabrication of two principle designs of a spiral spring-type applicator assembled from stacks of polymethylmethacrylate (PMMA) and polycarbonate (PC) sheets, respectively, by means of a simple double-sided adhesive tape. The applicators consist of the actuator and a guidance ring. Actuation can be simply performed by means of thumb pressure (TP). When the guidance ring is reached during actuation, the user receives haptic feedback. Figure 3 depicts an advanced design of this applicator configuration made from one piece, which was realized by commercial 3D printing (Shapeways, Eindhoven, The Netherlands).

#### 2.2.3. Experimental Evaluation of the Applicator-Assisted Insertion Method

We used three types of simple artificial models to emulate the spring-type behavior of fat and muscle and skin tissue of the inner forearm, whereas the different layers mimic together the stretchable top-layer (epidermis) of the skin: (1) a polymer foam (packaging material) of a thickness of 3.5 cm, followed by a layer of household paper tissue, household aluminum foil as a read-out layer to demonstrate penetration and a thin household PE-foil; (2) a ballistic gel received by mixing 20 g household gelatin (Dr. Oetker, Germany) with 147 cL water, yielding a concentration of 12%, household aluminum foil as a read-out layer to demonstrate penetration, and a Parafilm^®^ M top layer; (3) a layer of polydimethylsiloxane of 5 mm thickness, followed by the two layers of aluminum and PE foil.

The ballistic gel was prepared according to supplier instructions by mixing the gelatin sheets with water and gentle heating of the mixture under continuous stirring until all the gelatin was dissolved. After the mixture cooled down, it was placed in a refrigerator for 2 h. The obtained gel was reheated to remove air bubbles, and after cooling down it was placed in a refrigerator for 24 h for final solidification in a small plastic bowl.

#### 2.2.4. Evaluation of Puncture Efficiency

For back-light optical microscopy and image analysis of the resulting puncture hole shapes, we used an inverted Nikon Ti-E microscope and an Andor iXon ultra camera to collect images from the aluminum foil after the penetration experiment. For quantification of the puncture efficiency in the aluminum foil we used low-magnification back-light microscopy and imaging with a Zeiss Axioplan 2 microscope and a mobile phone camera.

#### 2.2.5. npMNA Images

For npMNA visualization we used either scanning electron microscopy (SEM) or a low-magnification hand-held lens and a mobile phone camera.

## 3. Results and Discussion

### 3.1. Indicative Measurement of Crack-Fracture Force

**Figure 4:**
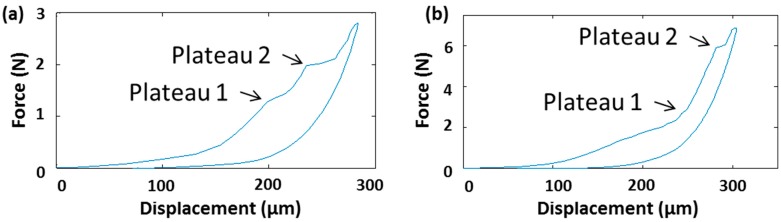
Examples of a force-displacement curve recorded by CSM microindentation on a single MN of a ceramic npMNA received from production run 2.6. (**a**) Force displacement curve clearly depicting the two plateaus of initial crack fracture (plateau 1) and at the full failure fracture (plateau 2); (**b**) curve with a less pronounced plateau 1.

For ceramic materials, particularly, the measurement of fracture forces is a research field on its own. Although the authors of this paper are not ceramic experts, we concluded that it is important to define a fracture force value for ceramic npMNAs to be able to compare this type of MNs with other MNAs described in the literature and use this value as an input parameter for the applicator design process. Therefore, we developed a method that determines material strength by microindentation onto a single MN. The method can be used to emulate the worst case scenario of device failure during self-administration of an MNA patch. Since method development is still on-going, we have not yet been able to statistically validate the method from a regulatory point of view. However, npMNAs yield single needle fracture force values well above the minimally required skin insertion force for microneedles with a tip radius below 12 µm. Ideally, two production runs utilizing the same process parameters should yield the same range of fracture forces by utilizing our fracture force measurement method. By means of an example, we compared two types of npMNAs prepared by the manufacturer: one npMNA (sample run named 2.6) made from the ceramic alumina-based nano-powder AKP 30 (Sumitomo, Tokyo, Japan), of which the slurry was mixed by the milling balls method on a rollerbench [12], and a second npMNA (sample run named 2.7) made from AKP 15 powder (Sumitomo), of which the slurry was mixed by automated stirring in a round-bottom flask. Both of these samples were sintered at 1450 °C in the same box oven but in different sinter batches. The generated force-displacement curves generally show two plateau types (Figure 4). We defined the onset of plateau 1 as the initial crack formation force and the onset of plateau 2 as the full failure fracture force, which leads to a complete loss of the MN tip. In some cases, the onset of plateau 1 is difficult to identify (Figure 4b); however, the results yielded an average initial crack fracture force at plateau 1 with indicative values of 1.823 ± 0.455 N for run 2.6 (*n* = 6) and 1.341 ± 0.443 N for run 2.7 (*n* = 9), respectively. Even with a safety factor of 3.6 [23], both these values are well above the estimated insertion force of 0.045 N for an individual MN penetrating skin tissue, assuming that the MN tip radius is below 12 µm.

### 3.2. npMNA Patch Applicator Design Study

**Figure 5:**
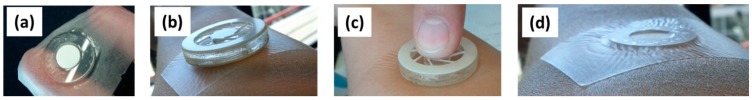
(**a**) npMNA patch assembled with guidance ring and adhesive; (**b**) MNA patch (emulated by a ceramic disc only) fixed with the guidance ring and the adhesive to the forearm of a volunteer with the first conceptual applicator atop; (**c**) The applicator assisting npMNA insertion pushing onto the piston; (**d**) The inserted npMNA.

Although it is the most profound manner to use thumb pressure (TP) actuation directly onto the base plate of a MNA patch [24], it is cumbersome if the MNA needs to be fixed to the skin by an adhesive (Figure 5a). It is possible that the adhesive already adheres to the surrounding skin area prior to providing intimate contact of the MNs residing into the skin layer. Consequently, this would lead to either total or partial drug delivery failure in the case of the ceramic npMNA patch. Although microincisions in the skin can still be made, the perceived npMNA drug delivery mechanism must then be described as a “poke and patch” approach rather than “poke and diffuse” [6]. Since we aim for the latter, we engineered a TP-actuated applicator-assisted insertion method to facilitate sufficient anchoring of the MNs in the skin by controlling the mechanical boundary conditions in the applicator-npMNA-skin interface.

A guidance ring (Figure 5a) already assists in anchoring MNs because it allows mainly vertical forces to be exerted onto the base plate of the npMNA after the adhesive is fixed to the skin. TP onto the npMNA base plate only may not spread the force distribution very uniformly across the base plate, and the adhesive may not be sufficiently deformed to attach against the sidewall of the inner diameter of the guidance ring firmly. Nevertheless, this concept minimizes shear forces onto the MNs and subsequently facilitates zero-MNs breakage.

**Figure 6:**
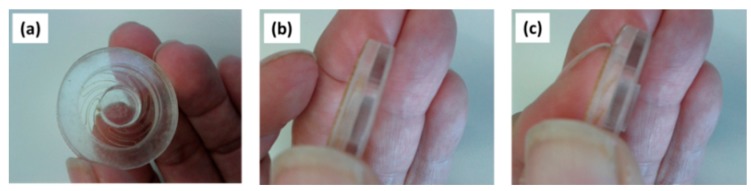
(**a**) Second conceptual applicator with a spiral-spring as a mechanical transducer for displacement of the piston; (**b**) Showing the applicator prior to actuation; (**c**) Showing the applicator in the actuated state.

Given the limitations of insertion by TP only, a pushing device (piston connected to a ring with a spring mechanism) atop the adhesive becomes handy (Figure 5b), which provides better control of the vertical displacement of the npMNA towards the skin, minimizing operational variations, independent of thumb or fingertip size, and includes haptic and visual feedback to the user (Figure 5c,d). This first applicator concept already provides the basic functions for insertion by a TP-actuated applicator that takes advantage of a unique bulging effect of the skin in the interface. However, our second conceptual design was realized with a spiral-spring applicator (Figure 6) for more robustness and a larger range of displacement. When exploring the latter concept in more detail, two issues remained: (1) Overcoming the bed-of-nail effect [29] by generating a high enough force distribution in the npMNA-skin interface via applicator-assisted insertion during TP onto the applicator piston; (2) Placement of the npMNA patch and adhesive in one handling step.

**Figure 7:**
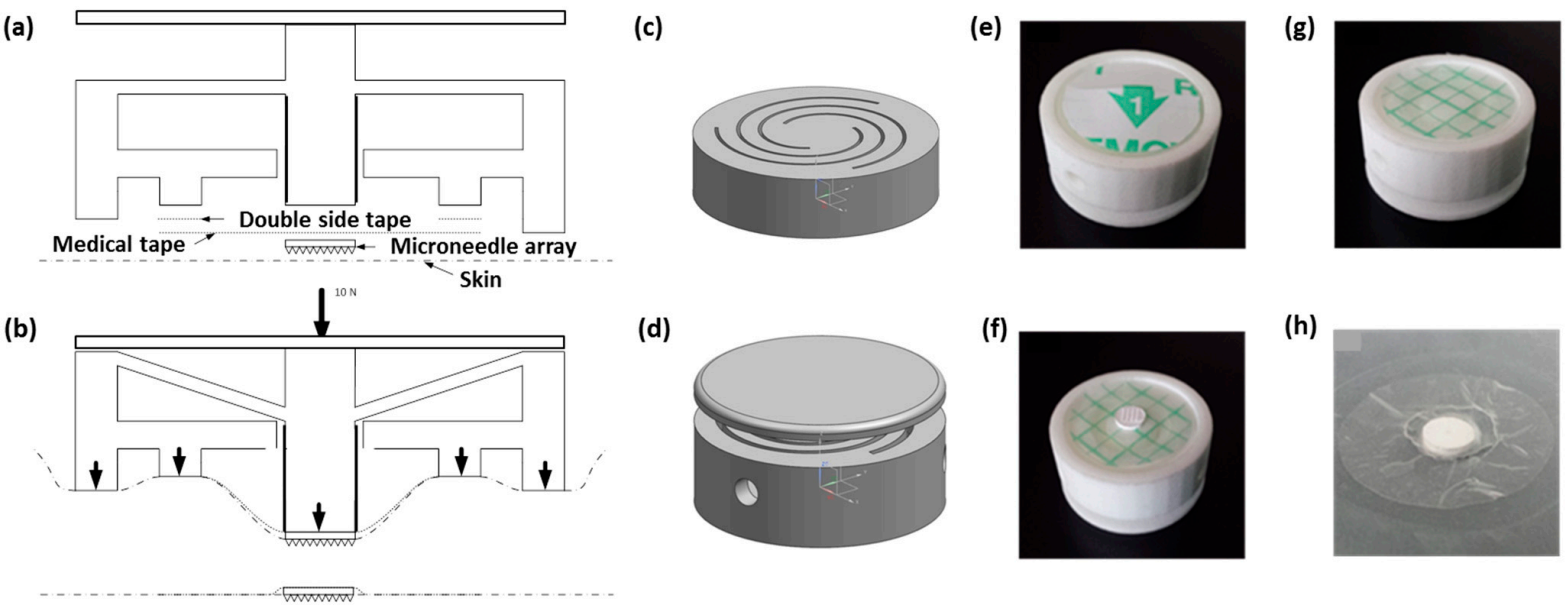
Design, realization and use of the 3D-printed spiral-spring applicator. (**a**) Schematic drawing of the applicator in the resting state; (**b**) Schematic drawing of the applicator in the pushed-in state; (**c**) 3D-design impression of the applicator without the push-plate; (**d**) 3D-design impression of the applicator with the push-plate; (**e**) Bottom view of the applicator assembled with an adhesive on the inner ring; (**f**) The same applicator as in (**e**) after removal of the protective foil of the adhesive and an npMNA attached atop; (**g**) Applicator with remainder of the back-side protective foil of the adhesive after insertion; (**h**) npMNA held in position by the adhesive atop a skin model.

Closely inspired by the conceptual spiral-spring design (Figure 6), an advanced 3D-printed applicator was fabricated in one piece by a commercial printing service (Shapeways), which consists of two concentric rings, the spiral-spring (Figure 7c) actuated piston, and a push-plate (Figure 7a,d). This design enables control of the bulging of the skin (Figure 7b), and hence the force distribution in the applicator-npMNA-skin interface upon pressing onto the pushing plate. Consequently, it neatly places an npMNA together with an adhesive on the skin for ease-of-handling (Figure 7e–h). This novel insertion method must also assist anchoring the npMNA by the adhesive in the skin. The applicator controls the boundary conditions in the applicator-npMNA-skin interface nearly independently of the operator. That is, when a user applies the npMNA patch, he or she will intuitively stop actuation as soon as the push-plate touches the applicator base ring—this maximal displacement refers also to the maximum load that can be exerted onto the MNs during TP-actuation of the piston.

We also carried out preliminary computational FEM analysis using Siemens’ NX 7.5 CAD-FEM program. A new material was inserted in the simulation package with a Young’s modulus of 12.5 kPa and a Poisson ratio of 0.48 (ballistic gel), which is similar to mechanical properties of human skin.

**Figure 8:**
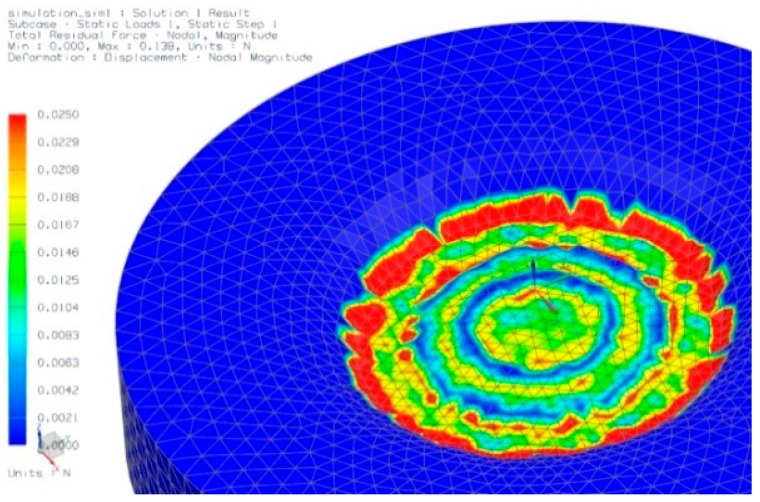
Simulation result of the bulging effect in the gel underneath of the applicator.

In a first approximation, we kept the material properties in the model layer isotropic. Establishing the optimal combination of parameters (*e.g.*, dimensions of the concentric ring structure) was done by trial and error. These simulations provided us with more insight on the required force distribution during actuation of the piston and the geometrical dimensions of the applicator. We designed the applicator to be fit for providing an insertion force of approximately 17 N. The simulation result confirms the bulging effect of the skin, and if a load is simulated on the piston, a reaction force of the skin layer can be derived (Figure 8). By means of these type of simulations, the design of the applicator-assisted insertion method can be further optimized.

### 3.3. Testing the Penetration Efficiency of the npMNA Insertion Method

**Figure 9:**
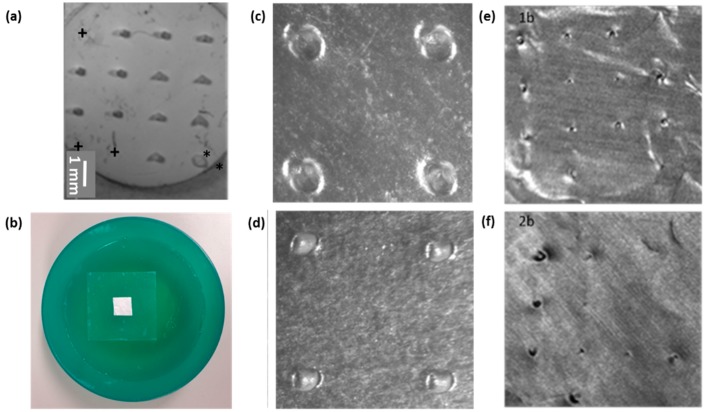
Penetration study with a SN-4×4-D300 npMNA on ballistic gel as a skin model. (**a**) Overview image of the used npMNA, * shows a defect instead of a MN, + indicates missing MNs prior to penetration into the skin model; (**b**) Ballistic gel with the aluminum foil and the parafilm atop; (**c**) Imprints into parafilm with the applicator; (**d**) Imprints into parafilm without the use of the applicator; (**e**) overview of corresponding puncture holes showing 12 out of 12 holes in the aluminum foil with the applicator; (**f**) corresponding puncture holes showing 4 out of 12 holes in the aluminum foil without the use of the applicator.

For our initial insertion tests comparing TP onto the npMNA base plate only with our new TP-actuated applicator-assisted method, we used an SN-4×4-R300 npMNA configuration, unfortunately already showing some defects prior to the insertion test (Figure 9a). For insertion into the ballistic gel skin model, which was prepared according to the instructions in the method in Section 2.2 and subsequently poured into a small, half-circular-shaped plastic bowl (Figure 9b), the parafilm had to be larger in surface area than the applicator in order to be able to mimic stretching of the skin. The layer of parafilm has approximately the same thickness as the epidermis of human skin and evidently shows imprints due to insertion of the MNs. TP onto the npMNA base plate only showed fewer pronounced imprints (Figure 9d) than with applicator-assisted TP actuation (Figure 9c).

Puncture holes in the aluminum foil provided us with a clear indicator for quantification of the puncture efficiency, depicting an efficiency 3 times higher for applicator-assisted TP actuation with 12/12 puncture holes (Figure 9e) versus 4/12 puncture holes (Figure 9f).

**Figure 10:**
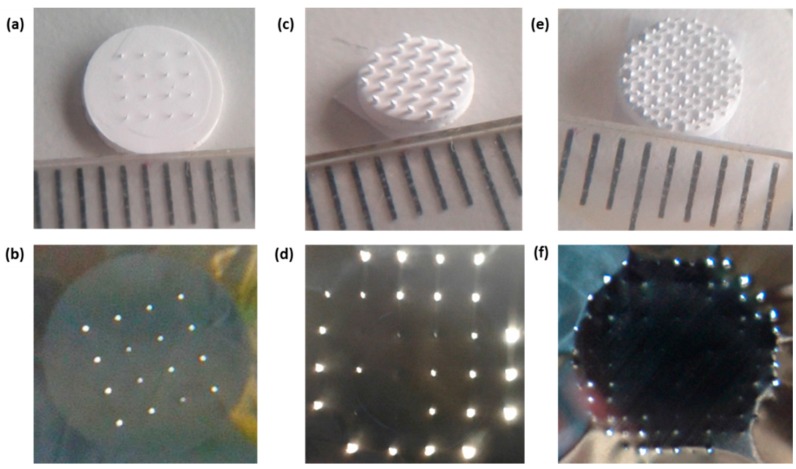
Overview images and corresponding back-light images of 3 different types of npMNA on foam as a skin model. (**a**,**b**) SN-4x4-R100; (**c**,**d**) SN-fc-D300; (**e**,**f**) G4-fc-R200.

The puncture holes in the aluminum foil are very distinct and were visualized by optical microscopy in more detail using a simple foam skin model representing a skin being more difficult to penetrate. Using this foam model in conjunction with our applicator to evaluate puncture performance of different npMNA designs provides less defined mechanical clamping conditions during insertion then in the case of the ballistic gel but demonstrates that the puncture efficiency is clearly influenced by the npMNA configuration (Figure 10). This simple model confirmed the findings in the literature [30] that MNs with a small MN base diameter and a relatively large inter-MN spacing, represented here by the SN-4x4-R100 design with 320 µm MN length (Figure 10a), punctures the skin model easily while puncture for MNs with a larger MN base diameter and a denser configuration, represented here by the SN-fc-D300 design with a MN length of 560 µm (Figure 10c), is much more difficult. Furthermore, we found that the SN-fc-R300 npMNA clearly outperforms the high-density G4-fc-R200 npMNA with a MN length of 200 µm, most likely because of the total microneedle length and the larger force per needle distributed by the applicator over a fewer number of microneedles in approximately the same surface area of the npMNA.

**Figure 11:**
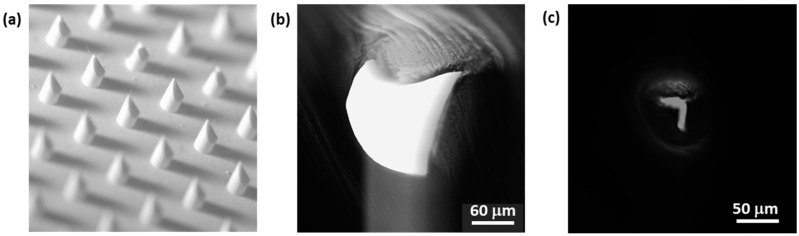
Detailed image analysis for a SN-fc-D300 npMNA on foam as a skin model. (**a**) Optical micrograph of MNs of the ceramic npMNA (Figure 10c) showing the knife-like shape of the tip; (**b**) Example of one of the large puncture holes (Figure 10d) showing the details of the cutting line corresponding with the tip shape presented in (**a**), scale bar = 60 µm; (**c**) Example of one of the smallest puncture holes (Figure 10d) still showing the distinct features of the cut by the unique shape of the MN-tip presented in (**a**), scale bar = 50 µm.

Since we previously performed penetration and human skin delivery experiments with SN-4×4-D300 and G4-fc-R200 npMNAs [6], we investigate these types of designs for their puncture performance in more detail by back-light microscopy in our simplified skin models. We firstly used the foam skin model for insertion and subsequently compared it with a much stiffer skin model using a 5 mm thick layer of PDMS instead of the foam. Based on availability, we used a SN-fc-D300 npMNA with 31 MNs for insertion tests by the TP-actuated applicator into the foam model, which demonstrated an average puncture efficiency of 81% ± 6.4%. Different puncture shapes and sizes can be detected corresponding to the force distribution, which originates at the applicator-npMNA-skin interface due to the clamping conditions during TP-actuation of the push-plate of the applicator (Figure 10b,d,f). Puncture holes have a very distinct shape (Figure 11b,c), agreeing with the knife-like tip geometry of this specific npMNA (Figure 11a). For SN-4×4-D300 using an impact-insertion applicator on dermatomed 600 µm thick human skin supported by a relatively hard styrofoam plate, an insertion test method previously published by Verbaan *et al.* [31], the penetration efficiency was 100% [6]. Such experimental conditions may resemble optimal but non-realistic clamping conditions during self-administration of an MNA patch. Furthermore, SN-4×4-D300 is a less challenging npMNA design than the SN-fc-D300, with 31 compared to 16 MNs in a similarly-sized base plate area. For the even more challenging G4-fc-R200 npMNA configuration, the penetration efficiency on foam was 24% (Figure 10f), or even lower; however, pressing an npMNA of this configuration onto the harder PDMS skin model, puncture efficiency was 80%, which is similarly high as in the case of using the impact applicator on human skin with a real skin penetration efficiency of 84.5% ± 3.6% [6] for this specific npMNA configuration. On the PDMS skin model, however, our TP-actuated applicator does not form a conformal interface with the skin model and the push-plate cannot be fully pressed down any more due to the much higher stiffness of the PDMS. Since we have only limited control of the actuation force being distributed in the case of pressing npMNA into the foil atop of PDMS, it is a less-preferred model for the further optimization of the TP-actuated applicator-assisted insertion method compared to either the foam or the gel model. On PDMS, the mechanism of insertion is actually similar to the case where one would press against the base plate with a pen-like device [29]. However, the direction of load application in using our TP-actuated applicator is still better-defined compared to a pen-like device, which has a much higher rotational freedom during handling.

We finally compared different G4-designs using a G4-fc-R200, a G4-3×3-R200, and a G4-3×3-R25 npMNA, respectively. We hypothesized that a more slender MN with a triangular base geometry would form a smaller contact area with the skin and hence result in a higher penetration performance than for MNs with a quarter circle base geometry of the same MN length. Using the foam model, both G4-3×3-R200 and G4-3×3-R25 actually showed very poor penetration efficiencies of 14% and 11%, respectively, and a significant difference was not noticeable between these two designs. Using the more extreme conditions of PDMS as skin model, however, we found that both designs were equally fit to puncture the aluminum foil at a rate of 100%.

**Figure 12:**
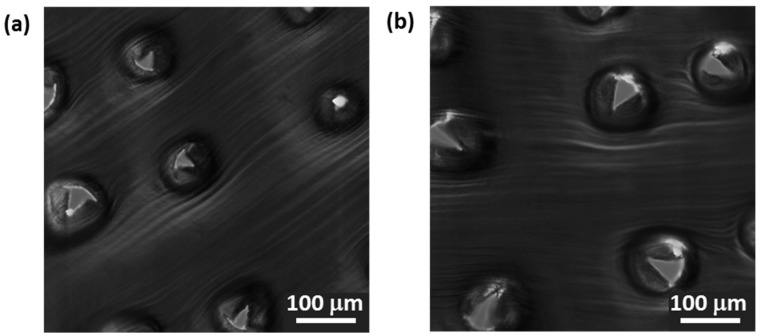
Optical micrograph analysis for G4-npMNA on PDMS as skin model. (**a**) Detailed image of G4-3×3-R200 puncture holes; (**b**) Detailed image of G4-3×3-R25 puncture holes. Scale bar = 100 µm.

**Figure 13:**
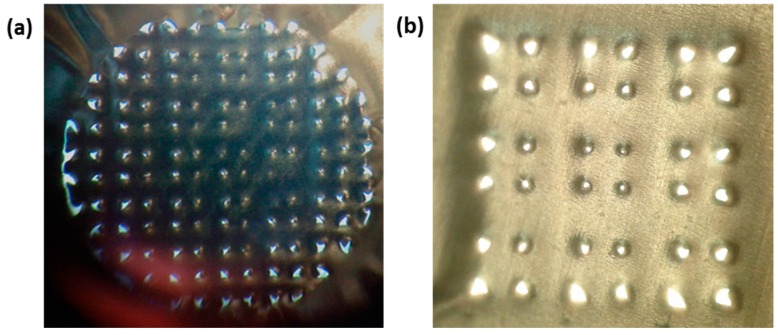
Evaluation of puncture efficiency for insertion of npMNAs in aluminum foil on PDMS as skin model. (**a**) Overview image of the distribution of puncture holes and imprints for G4-fc-R200 (Figure 10e); (**b**) Overview image of the distribution of puncture holes for G4-3×3-R200 depicting clearly smaller holes towards the center of the array.

More advanced back-light microscopy on the individual puncture hole shapes did not reveal specific differences between the two designs (Figure 12a,b). Therefore, we cannot confirm the above hypothesis under these conditions. On the other hand, it was clearly visible that there was a distinct difference in puncture hole shapes per array, with larger holes at the circumference of the array *versus* significantly smaller holes in the center of the array, as shown for the G4-3×3-R200 in Figure 13b.

In the case of using a G4-fc-R200 on the PDMS model, puncture efficiency yielded 80% (101/126 MNs), and although the effect of having larger holes in the circumference *versus* smaller holes in the center is less pronounced for this design, it is still visible (Figure 13a).

From a manufacturing point of view, a slightly larger tip shape with fewer MNs per patch seems favorable. From a drug delivery point of view, however, the choice for either of the npMNA configurations is not an obvious one and needs to be addressed more carefully in a pharmaceutical setting.

The variety of existing npMNA configurations tested by the new TP-actuated applicator-assisted insertion method showed a higher penetration efficiency than with TP onto the base plate of the npMNA directly. We find very distinct puncture hole shapes corresponding to the unique tip geometry of this type of MNAs; however, puncture efficiency for all these layouts mainly corresponds to the definition of the boundary condition in the applicator-npMNA-skin interface. While our findings suggest that for all the tested npMNA configurations an optimized parameter space for the applicator design being used on human skin can exist, we suggest that the mechanical optimization of these mechanical boundary conditions should benefit from the use of simplified skin models in order to find the best match between applicator design and a specific npMNA geometry, which must also take the variations in npMNA base plate thickness, base plate diameter, total number of needles and their density in an array, MN length, and the resulting MN tip sharpness due to variations in the production process more carefully into account.

## 4. Conclusions

In this paper we discuss the insertion process of ceramic nanoporous microneedles by means of a novel mechanical applicator design. This development of two new methods facilitates the evaluation of the readiness of npMNA technology for patch technology. First, single microneedle crack-fracture forces were measured by means of a microindenter reaching values 10× higher than the required insertion force for a single microneedle to penetrate human skin. Further, we demonstrated that our new thumb pressure-actuated applicator-assisted insertion method motivates operator independence and enhances puncture efficiencies in challenging high-density designs at least with a factor of 3 compared to thumb pressure onto the npMNA base plate only.

Using three artificial skin models in this study, it was confirmed that puncture holes into aluminum foil can be reliably studied by back-light microscopy to evaluate the insertion process dependent on various design parameters given in a specific MNA configuration. We also found that puncture efficiency on top of stiff PDMS was similarly high to an impact insertion applicator on *ex vivo* skin and may therefore serve as a reference. However, either foam or gel are the preferred models for the optimization of the boundary conditions in our thumb-pressure actuated applicator-assisted insertion method since these are reflecting more challenging insertion conditions similar to human skin tissue on the forearm and suffice in the design process over animal skin. Our new insertion method allows us to benchmark different ceramic npMNA patch designs and npMNA quality, either in production or against other MNA technologies prior to exploiting such devices in cost-intensive *ex*- or *in vivo* pharmaceutical settings.

In conclusion, the 80% penetration efficiency found for the SN-fc-D300 npMNAs on foam by using our TP-actuated applicator-assisted insertion method is a proof-of-principle that ceramic npMNAs are fit for MN assembly into a patch for the self-administration of drugs and vaccines.
